# Construction of a weight-based seed sorting system for the third-generation hybrid rice

**DOI:** 10.1186/s12284-021-00510-y

**Published:** 2021-07-13

**Authors:** Jianxin Wu, Shijun Qiu, Menglong Wang, Chunjue Xu, Xing Wang Deng, Xiaoyan Tang

**Affiliations:** 1 Guangdong Provincial Key Laboratory of Biotechnology for Plant Development, School of Life Sciences, South China Normal University, 510631 Guangzhou, China; 2 Shenzhen Institute of Molecular Crop Design, 518107 Shenzhen, China

**Keywords:** hybrid rice, nuclear male sterility, weight-based seed sorting, amiRNA, *OsAGPL2*, *OsAGPS2*

## Abstract

**Background:**

The third-generation hybrid rice technology can be constructed by transforming a recessive nuclear male sterile (NMS) mutant with a transgenic cassette containing three functional modules: the wild type male fertility gene to restore the fertility of the mutant, the pollen killer gene that specifically kills the pollen grains carrying the transgene, and the red fluorescence protein (*RFP*) gene to mark the transgenic seed (maintainer). The transgenic plant produces 1:1 NMS seeds and maintainer seeds that can be distinguished by the RFP signal. However, the RFP signals in the partially filled or pathogen-infected maintainer seeds are often too weak to be detected by RFP-based seed sorting machine, resulting in intermingling of the maintainer seeds with NMS seeds.

**Results:**

Here we constructed a weight-based seed sorting system for the third-generation hybrid rice technology by silencing the genes encoding ADP-glucose pyrophosphorylase (AGP) essential for endosperm starch biosynthesis via endosperm-specific expression of artificial microRNAs (*amiRNA*s). In this system, the NMS seeds have normal endosperm and are heavy, but the maintainer seeds have shrunken endosperms and are light-weighted. The maintainer seeds can be easily and accurately sorted out from the NMS seeds by weight-sorting machines, so pure and fully filled NMS seeds are available.

**Conclusions:**

The weight-based seed sorting system shows obvious advantages over the RFP-based seed sorting system in accuracy, efficiency, and cost for propagation of pure male sterile seeds. These characteristics will significantly increase the value and transgenic safety of the third-generation hybrid rice technology.

**Supplementary Information:**

The online version contains supplementary material available at 10.1186/s12284-021-00510-y.

## Findings

Heterosis utilization is the most effective way to improve crop production. Currently, hybrid rice production uses the cytoplasmic male sterile (CMS) lines or photoperiod/thermo-sensitive genic male sterile (PTGMS) lines as female parent (Cheng et al. 2007). CMS lines are caused by abnormal mitochondrial (Mt) genes, and they are propagated by crossing with the maintainer line carrying normal Mt gene and identical nuclear genome (Chen and Liu 2014). The CMS hybrid seeds are produced by crossing with the restorer line carrying restorer (*Rf*) genes that can specifically inhibit the function of the aberrant Mt gene (Cheng et al. 2007; Chen and Liu 2014). However, because *Rf* genes exist in only a few germplasms, the great majority of genetic resources cannot be explored for heterosis, thus restricting the breeding of superior hybrids (Cheng et al. 2007). PTGMS lines are caused by recessive nuclear genes, and their male fertility is influenced by environmental conditions such as photoperiod and temperature (Cheng et al. 2007; Fan and Zhang 2018). PTGMS lines are propagated via self-pollination under conditions restoring the male fertility. Under conditions inhibiting the male fertility, PTGMS lines outcross with paternal lines to produce hybrid seeds (Cheng et al. 2007; Fan and Zhang 2018). PTGMS lines can cross with any plants with the wild type fertility gene, thus almost all rice germplasms can be explored for superior heterosis. However, because fertility of PTGMS lines is sensitive to environmental conditions, both propagation of PTGMS seeds and production of hybrid seeds require strict environmental conditions, and both are vulnerable to unpredictable environmental changes (Cheng et al. 2007). Nuclear male sterile (NMS) lines insensitive to environmental conditions are common in flowering plants (Shi et al. 2015). However, application NMS lines for hybrid production is restricted because pure male sterile lines cannot be produced. In 2006, DuPont-Pioneer devised Seed Production Technology (SPT) in maize by transforming the NMS mutant *ms45* with the wild type *MS45* gene linked with the maize α-amylase gene *ZmAA1* under a pollen specific promoter to disrupt pollen grains carrying the transgene, and the *RFP* gene under a seed specific promoter to mark the transgenic seeds (Albertsen et al. 2006). Self-pollination of the transgenic plant produced 50% male sterile seeds and 50% transgenic seeds that could be sorted out based on the red fluorescence. Since then, various systems similar to SPT have been constructed in maize and rice with different *NMS* genes and pollen-killer genes, but all use RFP as the seed sorting marker (Chang et al. 2016; Wu et al. 2016; An et al. 2019; Qi et al. 2020; Wang et al. 2020; Liao et al. 2021; Song et al. 2021).

RFP-based seed sorting requires machines of high precision and complicated designs for seed delivery, florescence excision, florescence detection, image acquisition, and seed sorting (Wu et al. 2016; Song et al. 2021). However, partially filled seeds or pathogen-infected seeds often have low florescence that cannot be detected by the machine, resulting in contamination of the male sterile seeds by the maintainer seeds. To circumvent the problem, cross-pollination of NMS lines by the transgenic lines was used to propagate the NMS seeds, however, this practice is labor-intensive and low yield.

**Fig. 1:**
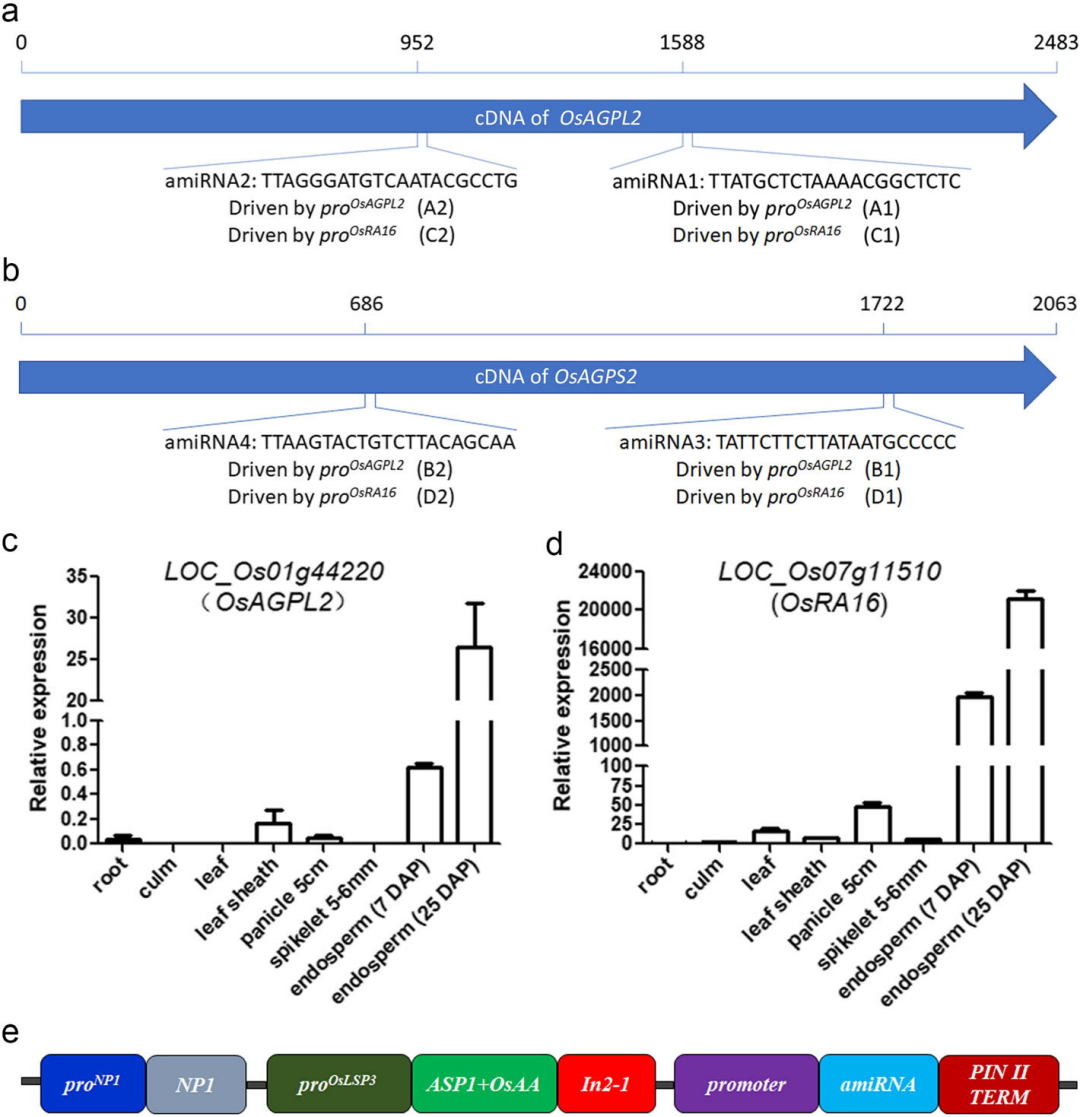
Design of the weight-based seed sorting system. (**a, b**) Target sites and the corresponding *amiRNA* sequences for silencing *OsAGPL2 *(**a**) and *OsAGPS2 *(**b**). Each *amiRNA* gene was driven by *OsAGPL2* promoter (*pro*^*OsAGPL2*^) and *OsRA16* promoter (*pro*^*OsRA16*^). The eight constructs were named as A1, A2, B1, B2, C1, C2, D1, and D2. (**c, d**) The spatial expression patterns of *OsAGPL2 *(**c**) and *OsRA16 *(**d**). The gene expression levels were determined by qRT-PCR with *Actin* gene as internal control. Data are shown as means ± SD (n = 3). DAP, day after pollination. (**e**) Diagram showing the three function modules. *Pro*^*OsNP1*^-Os*NP1* is for fertility restoration, pro^*OsLSP3*^-*ASP1* + *OsAA*-*In2-1* is for pollen inactivation, and promoter-*amiRNA*-*PINII TERM* is for silencing *OsAGPL2* or *OsAGPS2* gene. *In2-1* and *PINII TERM* are transcriptional terminators of the maize *In2-1* gene and the potato *PINII* (*Proteinase Inhibitor II*) gene, respectively (Chang et al. 2016)

**Fig. 2:**
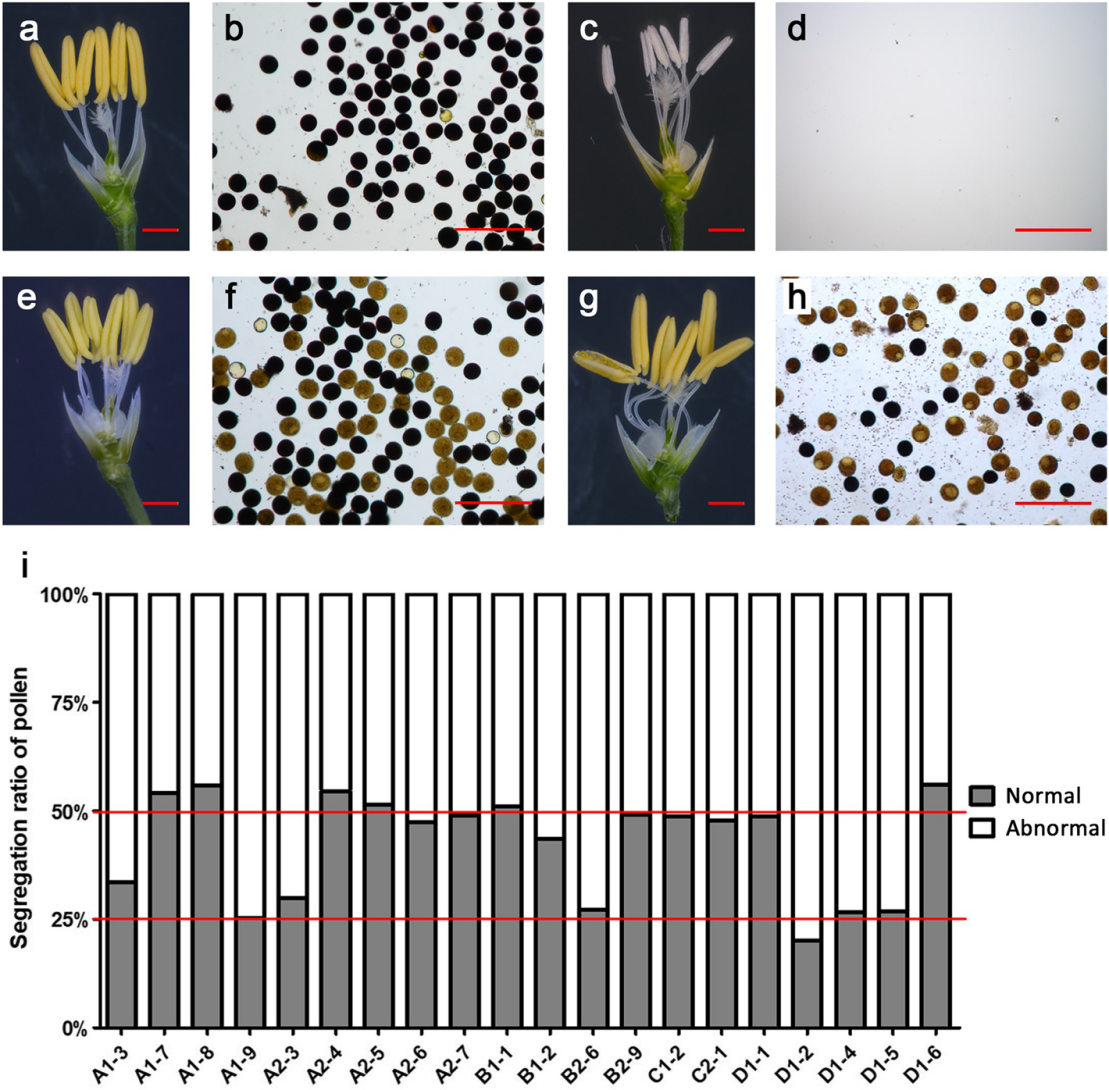
Anther morphology and pollen grains of the T_0_ transgenic plants. (**a-h**) Anthers and pollen grains from wild type (**a, b**), *osnp1* mutant (**c, d**), and representative transgenic plants A2-6 (**e, f**) and B2-6 (**g, h**). (**i**) The ratios of normal pollen grains to abnormal pollen grains from different transgenic plants. Pollen grains were stained with 1% I2-KI solution. Bar: (**a, c, d, g**) = 2 mm; (**b, d, f, h**) = 200 μm

**Fig. 3:**
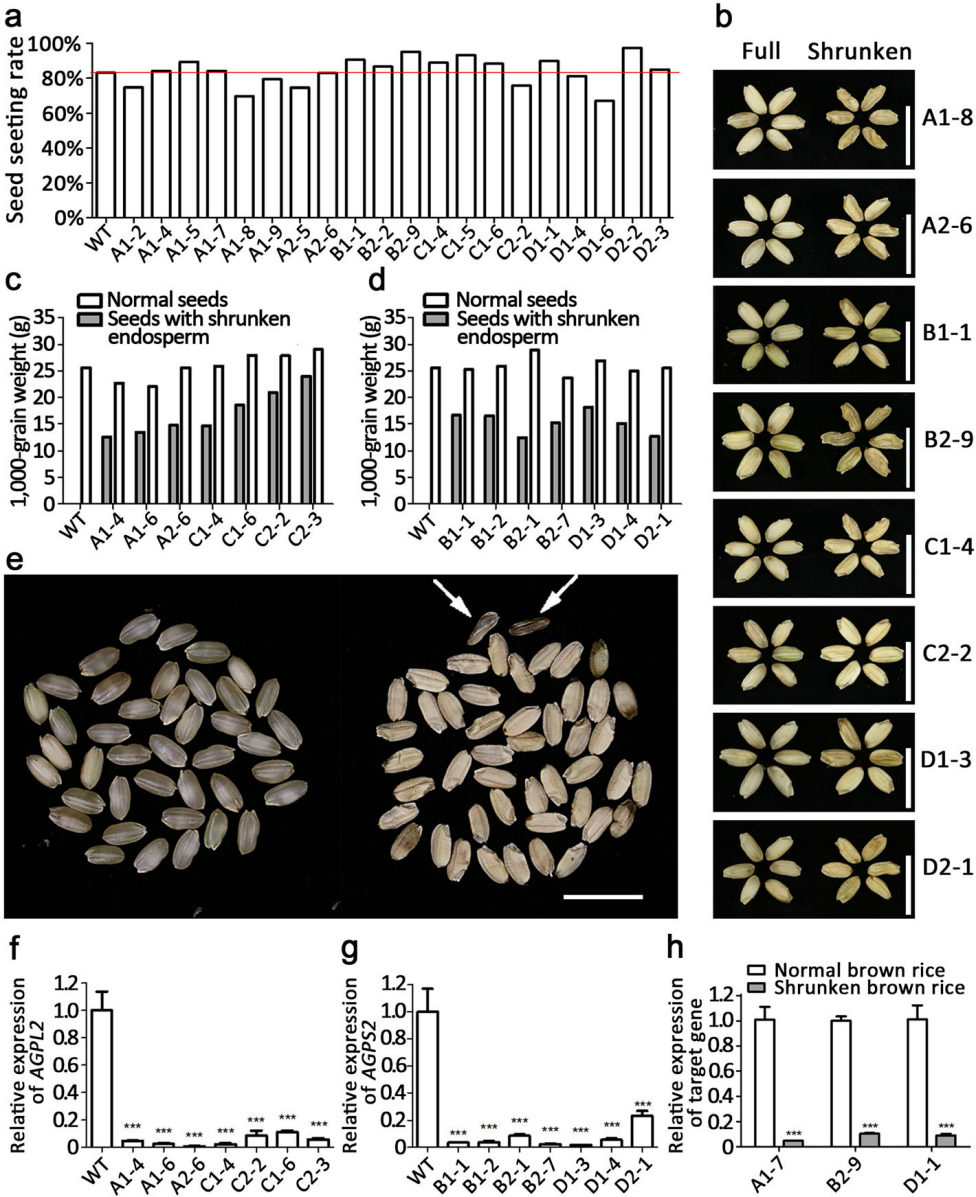
The morphology and weight of seeds from T_0_ transgenic plants. (**a**) The seed setting rates of the individual T_0_ transgenic plants. (**b**) The de-hulled grains from eight T_0_ transgenic plants. Grains with normal endosperm and grains with shrunken endosperm are shown. (**c, d**) 1000-grain weight of seeds from T_0_ transgenic plants silencing *OsAGPL2 *(**c**) and *OsAGPS2 *(**d.**) The seeds from each individual T_0_ plant were sorted by a weight sorter into the heavy-weight and light-weight groups. The 1000-grain weight was measured for each group. (**e**) The de-hulled heavy and light grains from A1-4. The arrows indicate two molded grains. (**f-h**) The *OsAGPL2* and *OsAGPL2* mRNA levels in the light and heavy seeds from the corresponding gene-silencing plants. The *OsAGPL2* and *OsAGPS2* transcript levels in the light seeds were compared with that in the wild type seeds (**f, g**) or that in the heavy seed (**h**). The transcript levels were determined using qRT-PCR with the *ubiquitin* gene as internal control. Data are shown as means ± SD (n = 3). Bar: (**b, e**) = 1 cm

Endosperm accounts for 89–91% of the total weight of rice grain, in which the majority component is starch (Lu and Luh 1991). ADP-glucose pyrophosphorylase (AGP) catalyzes the first committed step in starch synthesis in rice (Ohdan et al. 2005). AGP comprises two large subunits (AGPL) and two small subunits (AGPS), which are respectively encoded by four (*AGPL1, 2, 3, 4*) and two (*AGPS1, 2*) family members in rice (Lee et al. 2007). *AGPL2* and *AGPS2* are critical for starch accumulation in rice endosperm. Mutation or RNA interference of either gene causes shrunken endosperm and reduced grain weight, but the seeds are able to germinate and grow into normal plants (Lee et al. 2007; Tang et al. 2016; Wei et al. 2017). We proposed that grain weight manipulation through transgenic approach might generate a suitable marker for seed sorting for the third-generation hybrid rice technology. To test this hypothesis, we attempted to construct the weight-based sorting system by inhibiting the expression of *AGPL2* and *AGPS2* using artificial *miRNA* (*amiRNA*) (Warthmann et al. 2013) (Additional file 1: Methods). To ensure specific reduction of the grain weight to proper levels, we designed two *amiRNA*s targeting two different sites of each gene (Fig. 1a, b), each *amiRNA* was driven by two promoters of endosperm-specific genes, *LOC_Os01g44220* (*OsAGPL2*) (Fig. 1c) and *LOC_Os07g11510* (*Rice Allergen 16*, *OsRA16*) (Fig. 1d), respectively, resulting in eight recombinant *amiRNA* expression cassettes. We also devised a pollen-killer gene by constructing the rice α-amylase gene *OsAA* (*LOC_Os04g33040*) fusion with the amyloplastid targeting signal peptide ASP1 of α/β-hydrolase (*LOC_Os01g39800*) under the pollen-specific promoter of *OsLSP3* (*late-stage pollen-specific gene 3*) (Wang et al. 2020) (Fig. 1e). The eight recombinant *amiRNA* expression cassettes were individually linked with the pollen-killer gene and the male fertility gene *OsNP1* (*No Pollen 1*) (Fig. 1e), and transformed into *osnp1* mutant (Chang et al. 2016). Most of the transgenic plants showed 1:1 segregation of normal and abnormal pollen (Fig. 2e-f, i), implicating single transgene insertion. A few transgenic plants showed 1:3 segregation of normal and abnormal pollen (Fig. 2g-i), implicating two transgene insertions. The transgenic plants all displayed normal seed setting (Fig. 3a), but on the same plant, some of the seeds contained full endosperm while some contained shrunken endosperm (Fig. 3b). Seeds from the same T_0_ transgenic plant were sorted into two groups using a weight sorter that separates grains according to their weight. The 1000-grain weights for the seeds in the two groups showed a big difference (Fig. 3c,d). All the grains in the heavy-weight collection had normal endosperm; whereas in the light-weight collection, all grains had shrunken endosperm, and a few grains appeared to be molded (Fig. 3e). The light-weight grains showed severe reduction in *AGPL2* or *AGPS2* transcripts compared with the wild type grains and the heavy-weight grains from the same plants (Fig. 3f-h). These results indicated that all the constructs were capable of silencing the targeted genes, reducing the grain weight to various degrees.

**Fig. 4:**
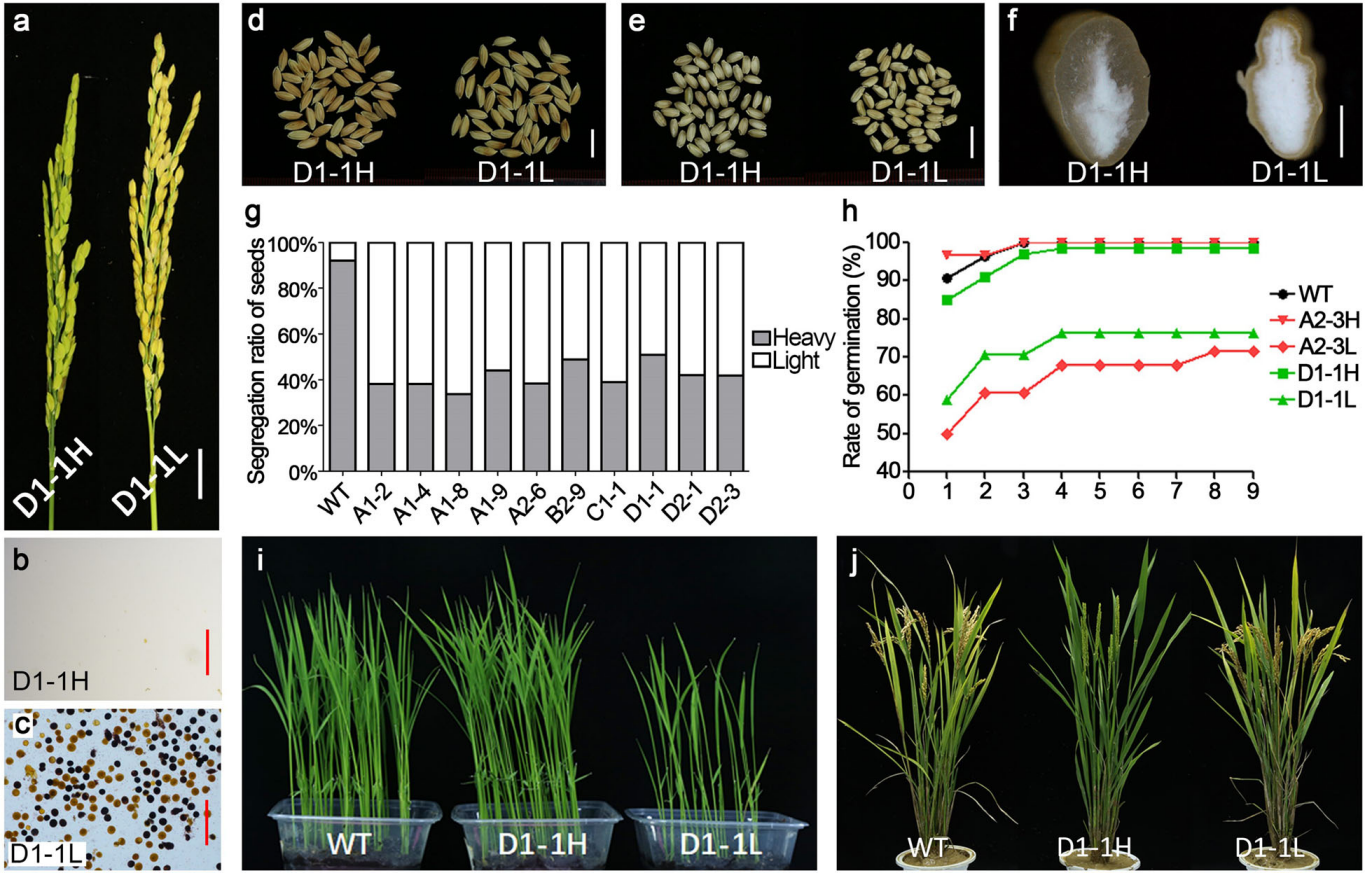
Characteristics of the T_1_ and T_2_ generations. Panicles (**a**) and pollen fertility (**b, c**) of the T_1_ plants derived from the heavy seed and light seed harvested from the D1-1 T_0_ transgenic plant. The T_2_ seeds harvested from the T_1_ generation plants were divided into heavy-weight and light-weight groups and examined for grain out-appearance (**d**), de-hulled grains (**e**), cut endosperms (**f**), rates of heavy and light grains (**g**), seed germination (**h**), vigor of seedling growth (**i**), and appearance of mature plants (**j**). Pollen grains were stained with 1% I2-KI solution. Results from D1-1 plant were shown as a representative. Bar: (a) = 2 cm; (b, c) = 200 μm; (d, e) = 1 cm; f = 1 mm

The heavy-weight grains (875 in total) from T_0_ plants were planted, and all showed male sterility (Fig. 4a,b). Plants from the light-weight grains showed 1:1 segregation of normal and abnormal pollen grains (Fig. 4a,c), and their grains also presented separation of full and partially filled grains despite similar out-appearance (Fig. 4d-f). After weight-based sorting, the ratios of heavy- and light-weight grains were analyzed. The wild-type plant had ~ 8% light-weight grains that were probably caused by environmental stresses, diseases and insects (Fig. 4g). Transgenic T_1_ plants derived from the light-weight seeds yielded 40–50% full grains (Fig. 4g). Some of the transgenic T_1_ plants yielded a bigger ratio of light-weight grains, probably because some of the grains were not well developed. The heavy-weight T_2_ seeds showed normal germination and seedling growth (Fig. 4h,i). The light-weight T_2_ seeds exhibited a lower seed germination rate (Fig. 4h), and seedlings were weaker before the five-leaf stage (Fig. 4i), but later became normal under regular field care (Fig. 4j). Approximately 20,000 T_2_ heavy-weight grains were planted for observation of male fertility, and all were sterile, indicating stable function of the *amiRNA* transgenes and accurate seed sorting.

The moderate weight-reduction of the maintainer seeds made the weight-based sorting system feasible to separate the maintainer seeds from male sterile seeds for the third-generation hybrid rice technology. Although the maintainer plants were slightly weaker at the early stage, later growth and seed setting were not affected. Compared with the RFP-based seed sorting system, weight-based seed sorting system has the following advantages. First, the RFP system cannot sort out the maintainer seeds with weaker fluorescence signal, resulting in intermingling of the maintainer seeds with the NMS seeds. This not only leads to transgene escaping into environment, the presence of maintainer lines in the male sterile lines during hybrid seed production is also a problem. However, the weight-sorting machine only keeps the full seeds. The partially filled male sterile seeds are also sorted out together with the maintainer seeds, which ensures high quality of the male sterile seeds without any maintainer seeds. Even though a small fraction of the male sterile seeds are immingled with the maintainer seeds, it is not problematic to production, because the male sterile plants can only be pollinated by the maintainer line to produce the male sterile seeds. Second, the RFP-based seed sorting machines require complicate designs and are expensive for manufacturing and maintenance. Currently, the most efficient RFP-based seed sorting machine can process only ~ 35 kg seeds per hour (Song et al., 2021). However, the weight-based sorting machines are common in seed industry and low cost. A regular machine can process 3000–6000 kg rice seeds per hour. Third, because the RFP-based seed sorting machines cannot sort out the maintainer seeds completely, production of male sterile seeds has to rely on cross-pollination of the male sterile lines by the maintainer lines, which requires separate planting of the parental plants and human-assisted pollination. These works are very laborious. However, weight-based sorting machines can sort out all the maintainer seeds efficiently, thus, direct seeding and self-pollination of maintainer lines can be used for propagation of the male sterile lines. This is suitable for mechanized production and will greatly reduce the labors and cost. Although the maintainer seeds showed a relatively lower germination rate (~ 70%), it is not a problem to production because most of the germinated seeds can grow into normal plants with normal seed setting under regular field care. Usually, a maintainer plant can produce ~ 2000 seeds; half of them are maintainer. Thus, the maintainer seeds are much more than needed for production even though they have a lower germination rate. The lower germination rate can be compensated by using more maintainers seeds during production. Besides, as various reduction of grain weight was observed in different maintainer lines, we are identifying an excellent maintainer line with a higher seed germination rate and good sorting performance.

In summary, we developed a weight-based seed sorting system for the third-generation hybrid rice technology by silencing the committed genes for starch synthesis via endosperm-specific expression of *amiRNA* genes. This system shows obvious advantages over the RFP-based seed sorting system in accuracy, efficiency, and cost for propagation of male sterile seeds. The application of this system will increase the transgenic safety and makes the third-generation hybrid rice technology more profitable. While it is proved here in rice, the same strategy can be applied to other crops as well to expand the utilization of heterosis. In rice, manipulation of grain weight can be achieved by regulating starch synthesis. For other crops, manipulating seed weight is also possible by regulating seed size if such gene is available.

## Supplementary Information

**Additional file 1:** Materials and Methods**. Table S1**. List of primers.

## Data Availability

The datasets supporting the conclusions of this article are included within the article and its additional files.

## Abbreviations

NMS: Nuclear male sterile
RFP: Red fluorescence protein
AGP: ADP-glucose pyrophosphorylase
AGPL: AGP large subunit
AGPS: AGP small subunit
amiRNA: Artificial microRNA
CMS: Cytoplasmic male sterile
Mt: Mitochondrial
PTGMS: Photoperiod/thermo-sensitive genic male sterile
Rf: Restorer
SPT: Seed Production Technology
ZmAA: Maize α-amylase
OsAA: Rice α-amylase
MS45: Male sterile 45
ASP1: Amyloplastid targeting signal peptide 1
OsNP1: Rice no pollen 1
