# Supplementary material for: Construction of a weight-based seed sorting system for the third-generation hybrid rice
Source: Rice (N Y). 2021 Jul 13;14:66. doi: 10.1186/s12284-021-00510-y (PMC8276899; doi:10.1186/s12284-021-00510-y)
Supplement: Supplementary file 1 — Additional file 1: Materials and Methods. Table S1. List of primers. [file 12284_2021_510_MOESM1_ESM.docx]

**Additional file 1. Materials and Methods**

**Plant growth and pollen fertility assay**

The *osnp1* mutant generated by Chang et al. (2016) in Wuyungeng (WYG) background was used for transformation of the weight sorting constructs. All the rice plants were grown in the patty field in Shenzhen with regular care. For pollen fertility analysis, rice anthers at the dehiscence stage were crashed in 1% I_2_-KI solution and photographed using a Nikon AZ100 microscope.

**RNA extraction and qRT-PCR**

Total RNA was extracted from various tissues using *TransZol* Up (TransGen Biotech) according to the manufacturer's instructions. Total RNA (500 ng) was used for cDNA synthesis using *EasyScript*^®^ All-in-One First-Strand cDNA Synthesis SuperMix (TransGen Biotech) according to the manufacturer's instructions. qRT-PCR was performed with the Applied Biosystems 7500 Real-Time PCR System using TB Green^®^ *Premix Ex Taq*™ II (Tli RNaseH Plus) (Takara). *Ubiquitin* (*LOC_Os03g13170*) were used as the internal control. All the primers for qRT-PCR are listed in Table S1.

**Design and cloning of *amiRNA*s, plasmid construction, and plant transformation**

The *amiRNA*s were designed according to the WMD3 system (http://wmd3.weigelworld.org/cgi-bin/webapp.cgi) targeting to the coding regions of *OsAGPL2* (*amiRNA1* and *amiRNA2*) and *OsAGPL2* (*amiRNA3* and *amiRNA4*). The *amiRNA* DNA fragments for plasmid construction were obtained using overlapping extension PCR according to the WMD3 instruction. All the primers for plasmid construction are listed in Table S1.

The *OsLSP3* promoter was obtained by PCR with the WYG genomic DNA as template and LSP3-F and LSP3-R primers. The PCR product and pCambia1300 plasmid were digested with *EcoR*I and *Kpn*I and purified from the gel. One Step Seamless Cloning kit (Aidlab biotechnologies Co., Ltd) was used for cloning of the *OsLSP3* promoter into the plasmid, resulting in the P1300-pro^LSP3^ construct.

Nipponbare genomic DNA was used as template for PCR-amplification of the amyloplastid targeting signal peptide *ASP1* with primers ASP1-F and ASP1-R and *OsAA* with OsAA-F and OsAA-R primers. pZhen18B (Chang et al., 2016) plasmid was used as template for PCR-amplification of terminator *In2-1* with In2-1-F and In2-1-R primers. The PCR products were purified and mixed together for overlapping extension PCR with ASP1-F and In2-1-R primers, resulting in the complete fragment of *ASP1-OsAA* carrying the *ASP1* signal peptide and the *In2-1* terminator.

The P1300-pro^LSP3^ construct was digested with *Kpn*I and *Hind*III, and the linearized plasmid was mixed with the complete fragment of *ASP1-OsAA* for ligation using the One Step Seamless Cloning kit (Aidlab biotechnologies Co., Ltd). The resulting plasmid 1300-L3AA was verified by sequencing.

The *PINII TERM* was PCR-amplified with pZhen18B plasmid as template and PINII-F and PINII-R primers. *miR4-1/2/3/4* fragments were PCR-amplified using *amiRNA1/2/3/4* DNA fragments as templates and miR-44220F and miR-R as primers. *miR1-1/2/3/4* fragments were PCR-amplified using *amiRNA1/2/3/4* DNA fragments as templates and miR-11510F and miR-R as primers. The PCR products were purified. *miR1-1/2/3/4* fragments and *PINII TERM* were mixed together with primers miR-44220F and PINII-R for PCR amplification of *44220-miR1/2/3/4*. *miR1-1/2/3/4* fragments and PINII TERM were mixed together with primers miR-11510F and PINII-R for PCR amplification of *11510-miR1/2/3/4*.

The promoter of *OsAGPL2* (*pro^OsAPGL2^*) was PCR-amplified using WYG genomic DNA and primer pair 44220pro-F/R. The promoter of *OsRA16* (*pro^OsRA16^*) was PCR-amplified using WYG genomic DNA and primer pair 11510pro-F/R. The construct 1300-L3AA was digested with *Hind*III and mixed with *pro^OsAGPL2^* and *44220-miR1/2/3/4* for ligation with the One Step Seamless Cloning kit (Aidlab biotechnologies Co., Ltd), resulting in constructs of 1300-L3AA-41/42/43/44. The construct 1300-L3AA was digested with *Hind*III and mixed with *pro^OsRA16^* and *11510-miR1/2/3/4* for ligation with the One Step Seamless Cloning kit (Aidlab biotechnologies Co., Ltd), resulting in constructs of 1300-L3AA-11/12/13/14. All the constructs were verified by sequencing.

*OsNP1* was PCR-amplified from pZhen18B plasmid (Chang et al., 2016) using OsNP1-F and OsNP1-R primers. The 1300-L3AA-41/42/43/44/11/12/13/14 constructs were digested with *EcoR*I and mixed with *OsNP1* for ligation with the One Step Seamless Cloning kit (Aidlab biotechnologies Co., Ltd), resulting in the final constructs of A1/A2/B1/B2/C1/C2/D1/D2. All the constructs were verified by sequencing and transformed into the *osnp1* mutant calli using the *Agrobacterium* mediated transformation.

**Table S1. Primers used for this study**

| Name | Sequence | Function |
| --- | --- | --- |
| LSP3-F | GAAACAGCTATGACATGATTACGAATTCGGGTGACGGCAGGTACT | Amplication of *OsLSP3* promoter |
| LSP3-R | GAGGATCCCCGGGTACCGAAAACCTTCTATGTGTCGTCT |  |
| ASP1-F | CACATAGAAGGTTTTCGGTACCATGCAGCCACTGGTGAG | Amplication of *ASP1* signal peptide |
| ASP1-R | ATCTGGCCCATTACCACTGCCCTTTGTTCCA |  |
| OsAA-F | GGCAGTGGTAATGGGCCAGATGGTTTCG | Amplication of *OsAA* |
| OsAA-R | GTCTTCCACCGATTCAACCTCCAAGAATGCT |  |
| In2-1-F | TTGGAGGTTGAATCGGTGGAAGACCACTC | Amplication of *In2-1*terminator |
| In2-1-R | CGACGGCCAGTGCCAAGCTTGGCGCGCCAATTC |  |
| amR1-1 | agTTATGCTCTAAAACGGCTCTCcaggagattcagtttga | Construction of *amiRNA1* |
| amR1-2 | tgGAGAGCCGTTTTAGAGCATAActgctgctgctacagcc |  |
| amR1-3 | ctGAGAGGCGTATTAGAGCATAAttcctgctgctaggctg |  |
| amR1-4 | aaTTATGCTCTAATACGCCTCTCagagaggcaaaagtgaa |  |
| amR2-1 | agTTAGGGATGTCAATACGCCTGcaggagattcagtttga | Construction of *amiRNA2* |
| amR2-2 | tgCAGGCGTATTGACATCCCTAActgctgctgctacagcc |  |
| amR2-3 | ctCAGGCCTATAGACATCCCTAAttcctgctgctaggctg |  |
| amR2-4 | aaTTAGGGATGTCTATAGGCCTGagagaggcaaaagtgaa |  |
| amR3-1 | agTATTCTTCTTATAATGCCCCCcaggagattcagtttga | Construction of *amiRNA3* |
| amR3-2 | tgGGGGGCATTATAAGAAGAATActgctgctgctacagcc |  |
| amR3-3 | ctGGGGGGATTTTAAGAAGAATAttcctgctgctaggctg |  |
| amR3-4 | aaTATTCTTCTTAAAATCCCCCCagagaggcaaaagtgaa |  |
| amR4-1 | agTTAAGTACTGTCTTACAGCAAcaggagattcagtttga | Construction of *amiRNA4* |
| amR4-2 | tgTTGCTGTAAGACAGTACTTAActgctgctgctacagcc |  |
| amR4-3 | ctTTGCTCTAACACAGTACTTAAttcctgctgctaggctg |  |
| amR4-4 | aaTTAAGTACTGTGTTAGAGCAAagagaggcaaaagtgaa |  |
| miR-44220F | GGGACTTTGAGTACCCTCGGATCCCAGCAGCAGCCACAGCAAA | *amiRNA* fusion with *PINII* TERM |
| miR-11510F | TTTCTGCAACAAAAATTCGGATCCCAGCAGCAGCCACAGCAAA |  |
| miR-R | TACCTACGCGTTCGAATCGGTACCGCTGCTGATGCTGATGCCAT |  |
| PINII-F | ACCGATTCGAACGCGTAGGTACCAC |  |
| PINII-R | ACGACGGCCAGTGCCAAGCTTGGCCGCATTCGCAAAACAC |  |
| 44220pro-F | CTCGAATTGGCGCGCCAAGCTTTATTGAGCTTTGTGA | Amplication of *OsAGPL2* promoter |
| 44220pro-R | TCCGAGGGTACTCAAAGTCCCAGACA |  |
| 11510pro-F | TCGAATTGGCGCGCCAAGCTTCATCAAGCCCATTTTGCCGA | Amplication of *OsRA16* promoter |
| 11510pro-R | TCCGAATTTTTGTTGCAGAAAATCTTAACT |  |
| OsNP1-F | GGAAACAGCTATGACATGATTACGAATTCTCAATTGAAGAATTTACCATTTGTC | Amplication of *OsNP1* |
| OsNP1-R | TCAGTACCTGCCGTCACCCGAATTCAATCCAGCCATATATATTTGTTGC |  |
| 11510-qpcr-F | GTTCTCAGTGTTGCTTCTCGC | *LOC_ Os07g11510* (*OsRA16*) qRT-PCR |
| 11510-qpcr-R | GGTCTTGGTGGTGGTACTCC |  |
| L2-qpcr-F | AAGCACTGAGGAAGAGGTGC | *LOC_Os01g44220* (*OsAGPL2*) qRT-PCR |
| L2-qpcr-R | TTTCGGGAGGATTGTGTCCG |  |
| S2-qpcr-F | ACGACGTGAGGGCAAAAGAA | *OsAGPS2* qRT-PCR |
| S2-qpcr-R | CCGATGTTTGTTGCACCTGG |  |
| Ubiquitin-F | CAACCAGCTGAGGCCCAAGAA | *Ubiquitin* qRT-PCR |
| Ubiquitin-R | CCAGGGAGATAACAACGGAAGC |  |
